# Correlation between Hierarchical Structure and Processing Control of Large-area Spray-coated Polymer Solar Cells toward High Performance

**DOI:** 10.1038/srep20062

**Published:** 2016-01-28

**Authors:** Yu-Ching Huang, Cheng-Si Tsao, Hou-Chin Cha, Chih-Min Chuang, Chun-Jen Su, U-Ser Jeng, Charn-Ying Chen

**Affiliations:** 1 Institute of Nuclear Energy Research, Longtan, Taoyaun 32546, Taiwan; 2 National Synchrotron Radiation Research Center, Hsinchu 30077, Taiwan; 3 Department of Chemical Engineering, National Tsing Hua University, Hsinchu 30013, Taiwan

## Abstract

The formation mechanism of a spray-coated film is different from that of a spin-coated film. This study employs grazing incidence small- and wide-angle X-ray Scattering (GISAXS and GIWAXS, respectively) quantitatively and systematically to investigate the hierarchical structure and phase-separated behavior of a spray-deposited blend film. The formation of PCBM clusters involves mutual interactions with both the P3HT crystal domains and droplet boundary. The processing control and the formed hierarchical structure of the active layer in the spray-coated polymer/fullerene blend film are compared to those in the spin-coated film. How the different post-treatments, such as thermal and solvent vapor annealing, tailor the hierarchical structure of the spray-coated films is quantitatively studied. Finally, the relationship between the processing control and tailored BHJ structures and the performance of polymer solar cell devices is established here, taking into account the evolution of the device area from 1 × 0.3 and 1 × 1 cm^2^. The formation and control of the special networks formed by the PCBM cluster and P3HT crystallites, respectively, are related to the droplet boundary. These structures are favorable for the transverse transport of electrons and holes.

Flexible and solution-processed polymer solar cells (PSCs) show promise as a potential photovoltaic technology because of their low cost, low weight, low energy consumption and easy fabrication by printing or coating techniques^1^,^2^,^3^,^4^. Recently, the power conversion efficiency (PCE) of PSCs comprising low-band-gap polymers has approached ~10%, close to the level required for commercialization^5^. The large-area spray-coating technique is one of the main candidates for potentially up-scaling and mass-producing an organic photovoltaic (OPV) module^6^,^7^,^8^,^9^,^10^,^11^,^12^. The conventional spin-coating process has been extensively adopted to deposit small-area high-performance PSCs. Previous studies clearly showed that PCE is mainly determined by the bulk heterojunction (BHJ) nanostructure of the blend film (i.e., a bi-continuous interpenetration network of self-organized polymers and aggregated PCBM molecules governing the exciton dissociation at the interface and charge carrier transport to the respective electrodes)^13^,^14^,^15^,^16^,^17^,^18^. The phase-separated BHJ structure of a spin-coated PSC is initially formed by the film-coating process and then tailored by various treatments, such as thermal and solvent annealing, to improve the PSC performance^19^,^20^. However, very few studies have reported a correlation of the hierarchical BHJ structure with the spray-coating process.

In essence, the film structure is influenced by the deposition conditions, such as solution concentration, drying kinetics, solvent and operating temperature. However, the process by which spray-coated films are formed is quite different from that of spin-coated films. For example, the unique characteristics of the spray-coating process include (1) atomization (i.e., breaking up bulk liquids into μm-scale droplets), (2) impact of the atomized droplets on the substrate (spreading the droplets on the substrate) and (3) aggregation and solidification of the droplets controlled by drying kinetics^21^. Thus, the hierarchical structure and formation mechanism of the active layer in spray-coated polymer/fullerene blend films are different from those in spin-coated films. It is still unclear how hierarchically phase-separated nanostructures of spray-coated films are formed. Although large-area fabrication techniques control surface morphology more roughly than the spin-coating method, recent studies showed that the performance of OPV devices fabricated by large-area techniques could be improved to reach a PCE value comparable to that of spin-coated PSCs by the use of post-treatments designed to tailor the internal self-organized structure of the film^10^,^11^. In particular, the droplet boundaries inside the film, which degrade the transport of charge carriers and thus device performance, can be eliminated by post-treatment^12^. Information regarding how the phase-separated BHJ nanomorphology (or film morphology) of spray-coated films can be modified at multiple length scales by the various treatments is still not available. The relationship among fabrication, nanomorphology and photovoltaic performance for spray-coated films needs to be systematically explored. This relationship would play an important role in optimizing the fabrication of large-area OPV devices.

This study quantitatively investigated the hierarchical structure and related formation mechanism of spray-deposited blend films using grazing incidence small- and wide-angle X-ray Scattering (GISAXS and GIWAXS)^13^,^14^,^15^,^16^,^22^,^23^. In parallel, we also investigated spin-coated films as a reference. The comparison between structures fabricated by the spray-coating and spin-coating processes is of interest for the development of other large-area OPV fabrication methods. This study provides an in-depth understanding of how the spray-coating parameters control the hierarchical structure of films and insight into the phase separation, which is different from that extensively reported for the spin-coating method. To date, all published papers using the X-ray scattering technique have focused on PSCs prepared by spin-coating followed by various treatments. The BHJ structure formed in the PSCs depends critically on the coating process because of the different formation mechanisms. The X-ray scattering characterization of PSCs prepared by spray-coating followed by various treatments is reported for the first time in this study. The structural characterization includes (1) orientation distribution, relative crystallinity and lamellar structure of nano-scale edge-on and face-on P3HT crystallites, (2) size and spatial distribution of nano-scale PCBM clusters and (3) meso-scale structure of amorphous P3HT/PCBM domains. The formation of PCBM clusters involves mutual interactions with both the P3HT crystal domains and droplet boundaries. We further quantitatively investigate how the different post-treatments (thermal and solvent vapor annealing) tailor the hierarchical structure of the spray-coated films. Finally, the tailored BHJ structures are correlated with the photovoltaic properties of an OPV device.

## Results

### Nanoscale PCBM clusters and mesoscale PCBM/P3HT domains: GISAXS analysis

**Figure 1:**
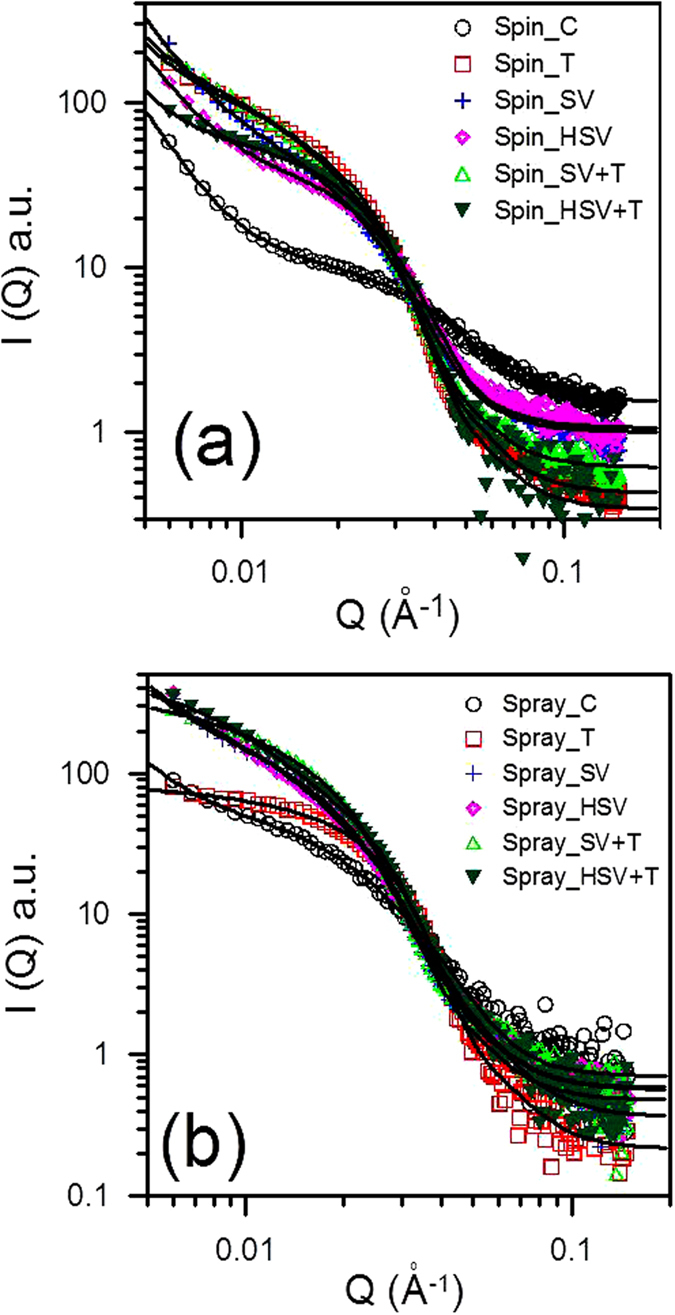
GISAXS profiles measured for (**a**) spin-coated and (**b**) spray-coated films with various post-treatments in comparison with the model-fitting intensities (solid lines).

The in-plane GISAXS profiles for all samples are shown in Fig. 1. According to previous studies^13^,^24^,^25^, the P3HT crystallites in the amorphous P3HT matrix in pristine P3HT film produced only weak GISAXS intensity. After the PC_61_BM molecules had been incorporated into the film (i.e., blend film), a broad peak or shoulder (at 0.02 ~ 0.03 Å^−1^) appeared in the medium-*Q* region of the GISAXS profiles. This broad peak is attributable mainly to PCBM clusters (formed from the aggregation of PCBM molecules). The intensity upturn in the low-*Q* region (0.006 ~ 0.01 Å^−1^) was attributed to the meso-scale phase, called the PCBM/P3HT amorphous domain^16^,^19^,^26^. This domain is composed of the amorphous P3HT polymer chains with the intercalated or dispersed PCBM molecules. The intensity upturn is mainly caused by the growing spatial distribution and increasing number density (or enhanced scattering contrast) of PCBM molecules dispersed or intercalated in the 3D network of the amorphous P3HT domain. The characteristic size of the meso-scale PCBM/P3HT amorphous domain can be modeled by the Debye-Anderson-Brumberger (DAB) equation using the correlation length, *ξ*. This DAB model is frequently used for model fitting of GISAXS intensity upturns (in the low-*Q* region) to interpret large-scale PCBM/P3HT amorphous structures.

To simultaneously resolve the structures of the PCBM clusters and the PCBM/P3HT amorphous domain, the measured GISAXS profiles can be modeled as given by^24^,^25^


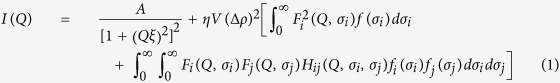


where *A* is a prefactor that is related to the electron density contrast in the DAB model. The first term in Equation (1) is the DAB model dominating at the low-*Q* region (less than 0.01 Å^−1^). The second term is the polydispersed sphere model containing Schultz size distribution with a hard-sphere interaction between PCBM clusters. The second term describes the broad peak in the medium-*Q* region of SAXS intensity contributed by the nanoscale PCBM clusters with an average cluster volume *V*. *F*(*Q*, *σ*_*I*_)^2^ is the form factor of the spherical PCBM cluster with a diameter *σ*_i_. *H*(*Q*, *σ*_*i*_, *σ*_*j*_) is the pair structure function describing the interaction between clusters with the Percus-Yevick approximation for hard-sphere interaction. The fitting parameters, *η*, *R* and *p*, are the volume fraction, mean radius and polydispersity of size distribution of the PCBM clusters, respectively. Δ*ρ* is the scattering length density contrast. All measured GISAXS profiles can be fitted^27^ well using the model of Equation (1), as shown in Fig. 1. The parameters in the different models are not affected by each other during the nonlinear least-squares calculation because their contributions are separable. The structural parameters *η*, *R*, *p*, *A*, and *ξ* extracted by the model fitting are listed in Table 1. Generally, the significant increase in the GISAXS intensity in the medium- and high-*Q* regions due to various post-treatments reveals the growth and increase in volume fraction of the PCBM clusters, which is related to the electron transport path in the BHJ structure.

### Mechanism and control of PCBM aggregation by spraying and post-treatments

From direct observation of a shoulder in the middle- and high-*Q* region of the GISAXS profiles (Fig. 1) and the model fitting results (Table 1), the volume fraction of PCBM clusters formed by the aggregation of PCBM molecules in the spray-casted film (Spray_C) is 6.7%. This value is much higher than the volume fraction of clusters in the spin-cast film (0.8% for Spin_C). The size of the PCBM clusters in the Spray_C film (*R* = 4.2 nm) is slightly larger than that in the Spin_C film (*R* = 3.2 nm). These sizes can be attributed to differences between the drying processes (or formation mechanisms). The fast drying kinetics of μm-scale droplets (during flight in air, impact on substrate, etc.) may cause a sharp change in the spatial distribution of PCBM concentrations and thus induce larger and more extensive PCBM aggregation compared to that of the spin process. However, the structures of meso-scale amorphous P3HT/PCBM domains are similar for both films.

After conventional thermal annealing, the volume fraction of nanoscale PCBM aggregation clusters in the spin-cast film increases significantly from 0.8 to 18.0%. The corresponding radius increases from 3.2 to 8.7 nm, which is consistent with the results of previous studies^19^,^25^. In contrast, the volume fraction of PCBM clusters in the spray-cast film does not change after thermal annealing. The radius of the PCBM clusters in the film increases from 4.2 to 7.7 nm. This result implies that thermal diffusion of PCBM molecules in the Spray_T film may be spatially limited or confined in a certain region (inside the boundary of each droplet). Thus, the Spray_T film allows the growth of PCBM clusters only in local regions. The meso-scale amorphous P3HT/PCBM domains in the Spray_T film are also confined. For the spray-coated films, the above quantitative GISAXS analyses (Table 1) reveal (1) the particular mechanism of PCBM aggregation induced by atomization and (2) the spatial PCBM distribution and PCBM aggregation confined inside the droplet boundary during thermal annealing.

In contrast, the results for the spray-cast films annealed by RT solvent vapor show that the volume fraction of PCBM clusters increases significantly to 12.5% (Table 1). In addition, the radius of the PCBM clusters in this vapor-treated film (Spray_SV) increases to 8.7 nm. These values show quantitatively that RT solvent vapor can dissolve or eliminate most of the droplet boundaries (reduce the hindrance) to enhance the long-range diffusion of PCBM molecules. This aggregation enhancement is also greater than that due to the effect of RT vapor annealing on the spin-cast film (Spray_SV film vs. Spin_SV film; see Table 1). Some of the details of the solvent-vapor-annealing effect on the spin-coated P3HT/PCBM blend film were reported in our previous study^20^. The structures of the meso-scale PCBM/P3HT amorphous domains for the Spin_SV and Spray_SV films are similar. Apparently, for the spray-coated films, solvent vapor annealing at RT can effectively enhance the formation of nanoscale PCBM clusters compared to thermal annealing. After additional thermal annealing is applied to the Spray_SV film (i.e., the preparation of the Spray_SV + T film), the volume fraction and radius of PCBM clusters increase further to 14.4% and 10.1 nm, respectively, compared to those of Spray_SV film (12.5% and 8.7 nm). This finding provides evidence that a thermal driving force can facilitate more PCBM diffusion and aggregation into larger clusters after the destruction of the droplet boundaries.

**Figure 2:**
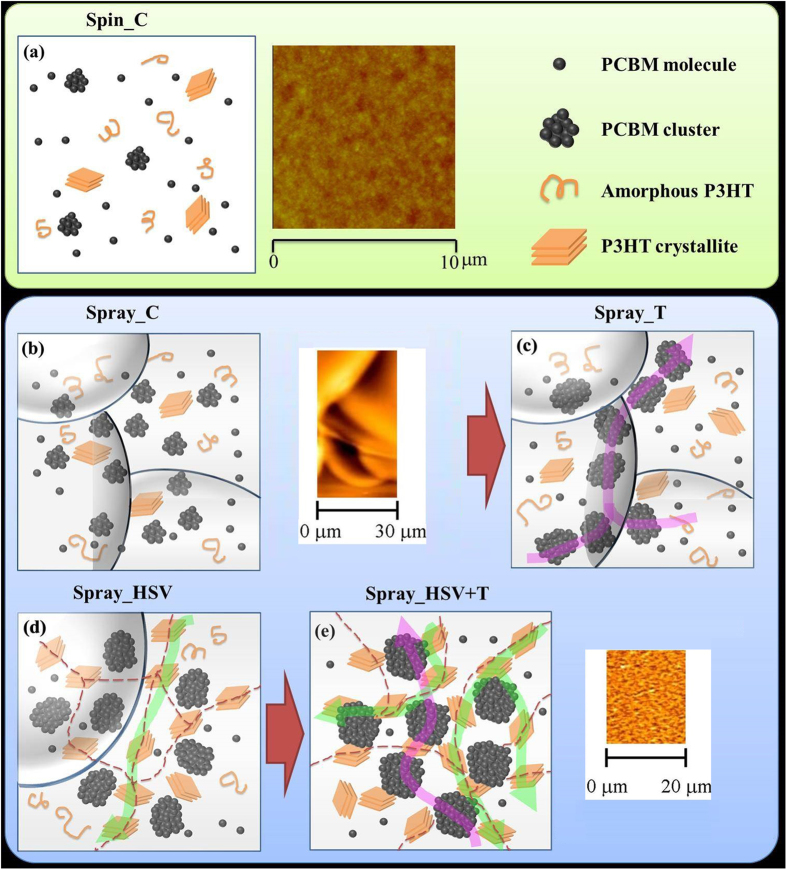
Schematics of the hierarchical BHJ structures controlled by various post-treatments. (**a**) Spin_C film with a low fraction of small PCBM clusters, (**b**) Spray_C film with a medium fraction of small PCBM clusters confined in the droplet boundaries, (**c**) Spray_T film with large aggregated PCBM clusters limited by the droplet boundaries (constructing into a PCBM network), (**d**) Spray_HSV film with few droplet boundaries and the network of P3HT crystallites formed with the elimination of the droplet boundaries. (**e**) Spray_HSV + T film with the interpenetrated networks formed by a large amount of P3HT crystallites and large PCBM clusters. The formation is closely related to the original droplet boundary. (**a,b,e**) include the representative AFM morphologies. These spray-coated BHJ structures reveal the mutual interaction between PCBM aggregation (nanoscale clusters) and P3HT crystallization confined in the droplet boundaries during phase separation. The hot solvent vapor and final thermal treatments eliminated the droplet boundaries and thus produced the related networks (as shown by dash lines) of P3HT crystallites or PCBM clusters. The favorable network paths for transporting electrons (purple arrow) and holes (green arrow) in the traverse direction were formed.

**Figure 3:**
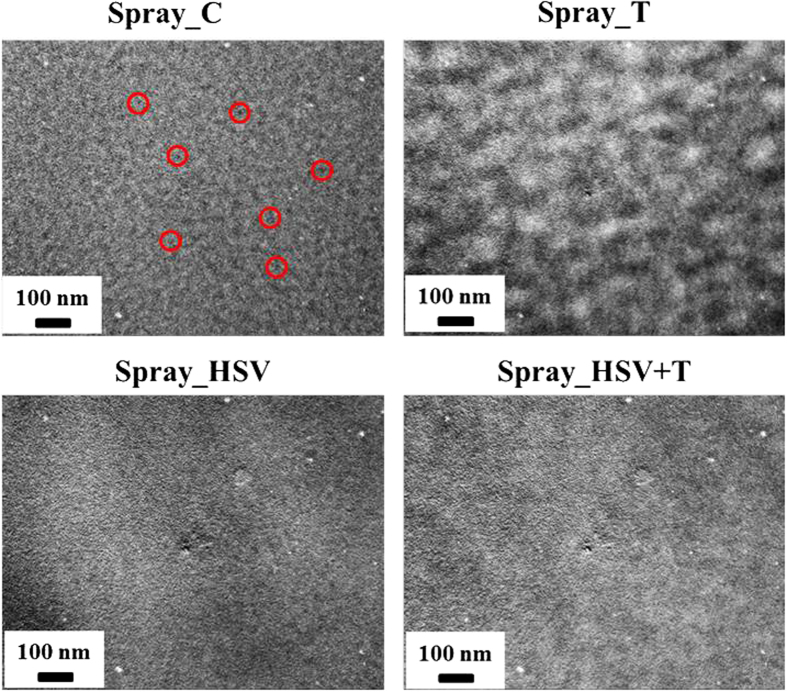
TEM images corresponding to the films of Fig. 2. (These common white spots in the TEM images are originated from the defect pixels of the detector used.)

In spray-cast film annealed by hot solvent vapor (Spray_HSV), the aggregation of PCBM clusters and amorphous P3HT/PCBM domains are similar to those in the Spray_SV film (Table 1). The data in this table show that hot solvent vapor annealing eliminates the droplet boundaries and thus facilitates PCBM aggregation in the same way as RT solvent vapor annealing. However, thermal annealing of the Spray_HSV film has a different effect from RT solvent vapor plus thermal annealing. The volume fraction of the PCBM clusters does not change (Spray_HSV + T vs. _HSV). The only change is that the size of the PCBM clusters increases from *R* = 7.6 to 9.6 nm. This result implies that the thermal growth of PCBM clusters is only a local behavior that is strongly confined in space or limited by the lack of long-range diffusion of PCBM molecules. This phenomenon can be explained by the fact that the PCBM molecules are confined by the high polymer crystallization or highly dispersed P3HT crystallites (evidenced by the corresponding GWAXS pattern, as discussed below) instead of the droplet boundaries. A mutual influence between P3HT crystallites and PCBM clusters has been reported in the literature^16^. The meso-scale amorphous P3HT/PCBM domains for both the Spray_SV + T and _HSV + T films are also confined to a reduced size compared to those of the corresponding spin-coated films (Table 1). Note that the confinement effect of the polymer crystal in the final thermal annealing of a two-step treatment for the spray-based film is larger than that for the spin-based film. It could be speculated that there exists a P3HT crystallite network distributed like droplet boundaries. The discrepancy between the mechanisms for the spin- and spray-coated films is summarized in Fig. 2. The details of Fig. 2, including AFM images, show (1) the mutual interaction between PCBM aggregation (nanoscale clusters) and P3HT crystallization confined in the droplet boundaries and tailored by hot solvent vapor annealing or further thermal annealing in the spray-coated BHJ structures; (2) the variation in droplet boundaries with various treatments; and (3) the droplet-boundary-limited network formed from the aggregation of P3HT crystallites or PCBM clusters. This type of P3HT network (or PCBM network) at the micro-meter scale has a remarkable advantage in facilitating the transport of charge carriers along the traverse directions (on an X-Y plane) compared to the well-reported BHJ structure of spin-coated PSC devices. The control of this characteristic network is important for the fabrication of large-area, high-performance PSC devices (as discussed below). The images of TEM corresponding to the schematic representation of Fig. 2 are shown in Fig. 3 for a rough comparison. The TEM image of the Spray_C film shows the black dots of ~6 nm in some local regions. These black dots may be PCBM clusters. The TEM image of Spray_T film shows the aggregation network of large PCBM clusters, which is consistent with the schematic representation of Fig. 2 predicted by our GISAXS analysis. This aggregation network of PCBM clusters is confined by the droplet boundary (invisible to TEM). Figure 2 shows that the Spray_HSV film has the large network formed from the aggregation of P3HT crystallites to disperse the PCBM clusters, which is consistent with the corresponding TEM image (the white zone may be P3HT network). The Spray_HSV + T film has large network formed from the large PCBM clusters in addition to the existence of original large P3HT network (in Fig. 2) is also consistent with the corresponding TEM image (white zone and black zone co-exist in a mixed way to represent the interpenetration network of P3HT and PCBM phases). The TEM images of the other films are in the supplementary information.

### Mechanism and control of P3HT crystallization by spraying and post-treatments

**Figure 4:**
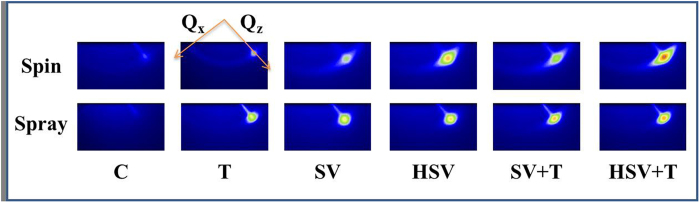
Two-dimensional GIWAXS patterns of Spin_C, _T, SV, HSV, SV + T and HSV + T films, and Spray_C, _T, SV, HSV, SV + T and HSV + T films. The bright spots represent (100) diffraction along the out-of-plane direction.

**Figure 5:**
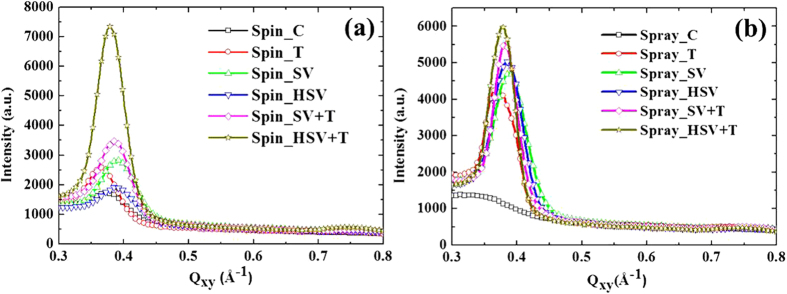
Out-of-plane GIWAXS profiles of (a) spin- and (b) spray-coated films with different treatments.

Figure 4 shows the 2D GIWAXS patterns for spin- and spray-coated films with different post-treatments. The out-of-plane GIWAXS profiles (reduced from the corresponding 2D patterns of Fig. 4) for the spin- and spray-coated films show the structural variation in edge-on P3HT crystallites due to the different treatments, as shown in Fig. 5. The positions and widths of major peaks in the GIWAXS profiles represent the inter-layer spacing and crystal size of the lamellar structure. In essence, the peak positions of spin- and spray-cast films are similar (~0.38 Å^−1^; corresponding inter-spacing of (100) layers: ~16.7 Å). The slight shift toward the low-*Q* region for both Spin_T and Spray_T films after thermal annealing reveals the ordering of side chains normal to the (100) plane. The lamellar structures of nano-scale, edge-on crystallite films formed from the spin and spray processes are the same and are consistent with previous studies^19^,^25^. However, the relative crystallinity and crystal growth due to thermal annealing of the spin-coated films (Spin_C vs. Spin_T films) are lower than those of spray-coated films with the same post-treatments (shown by the peak intensity in Fig. 4). This finding suggests that the droplet boundaries could not confine polymer crystallization on a large scale during thermal annealing.

**Figure 6:**
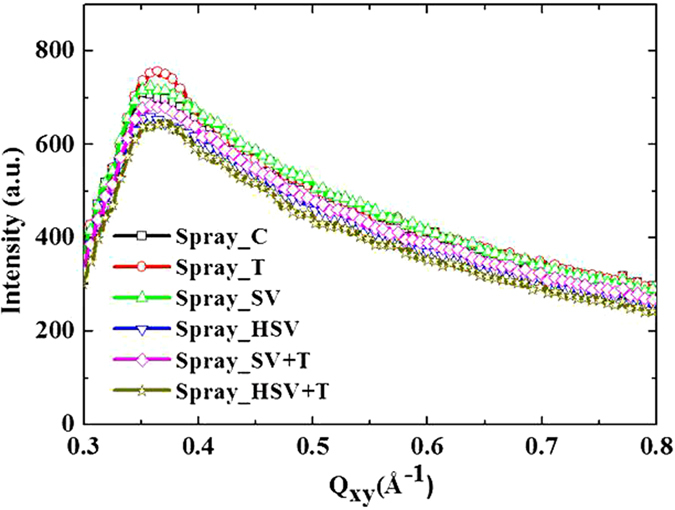
In-plane GIWAXS profiles of the spray-coated films with different treatments.

The Spray_HSV film shows that hot solvent vapor can enhance P3HT crystallization more effectively than in the Spray_SV films, although both films have similar PCBM structures. Herein, hot solvent vapor annealing has an exceptionally large thermal effect, enhancing polymer crystallization relative to PCBM aggregation. The enhancement factor (above 60%) for the spray-based film is much larger than that for the spin-based film (relative increase in the peak intensity of GIWAXS profiles; Fig. 5(a,b)). It can be speculated that the internal structure of a spray-coated film provides relatively favorable nucleation sites for polymer crystallization or a favorable spatial arrangement between PCBM clusters and P3HT crystallites for polymer crystal growth. Essentially, RT or hot solvent vapor annealing destroys the droplet boundaries, causing a large rearrangement of acceptor and donor components. Generally, polymer crystallization develops faster than PCBM aggregation durinig thermal annealing^28^. Moreover, the P3HT crystallinity of the Spray_HSV film after thermal annealing increases greatly compared to that of spin-coated films after the same treatments. It can be deduced that the fraction of P3HT crystallites in the Spray_HSV + T film relative to the other films is much larger than that in the Spin_HSV + T film. This finding provides evidence supporting the confinement effect on PCBM aggregation (in volume fraction) in the Spray_HSV + T film. Spray-coated films with different treatments have similar in-plane GIWAXS profiles (as shown in Fig. 6), revealing that their face-on crystallites (~20% of total P3HT crystallites) are not influenced by the post-treatments. The distribution of orientations of the major (edge-on) crystallites (indicated by the arc length of (100) spot in Fig. 4) and isotropic orientations of the other crystallites (ring shape) for the spin-coated films are more obvious than those for the spray-coated films. This result implies that the randomly oriented distribution of P3HT crystallites formed in the spray process is also caused by the limitations of droplet-related morphology.

### Relationship between tailored hierarchical structure and photovoltaic properties

Table 2 and Table 3 list the performance of spray-coated devices based on the above films (the corresponding I-V curves and the performance of spin-coated devices are shown in the Supplementary Information). For all films based on the spraying process, the relative intensities of the UV-visible absorption spectrum (Supplementary Information) are essentially consistent with the relative crystallinities measured by the integrated intensity of (100) peaks of the corresponding out-of-plane GIWAXS profiles. Generally, the high volume fraction and appropriately large size (15 ~ 20 nm) of PCBM clusters are favorable for electron transport without causing a loss of charge carrier separation (related to a reduction in the interfacial area between acceptor and donor components). In contrast, polymer crystallinity is closely related to the amount of exciton generation. As shown in Table 1, the volume fraction and size of PCBM clusters in the thermally annealed spray-coated film (Spray_T) are much lower than those in the Spray_SV film. Additionally, the Spray_T film has lower polymer crystallization (Fig. 4(b)). However, the PCE (1.8% for 1 × 0.3 cm^2^ device area; see Table 2) based on thermally annealed spray-casted film (Spray_T) is slightly larger than that based on the Spray_SV film (1.7% at the same area). It could reasonably be speculated that PCBM clusters in the Spray_T film distribute spatially throughout the connected boundaries of aggregated droplets, forming an effective 3D network and thus enhancing the electron transport. When the device area is extended to 1 × 1 cm^2^, PCEs based on Spray_C and _SV films are greatly reduced owing to the decrease in fill factor (Table 3). In contrast, the PCE based on the Spray_T film is slightly reduced, consistent with the speculation above. Note that the short-current J_SC_ (Table 3) of the 1 × 1 cm^2^ device based on the Spray_T film is relatively high, showing the effect of electron transport enhanced by the 3D network (Fig. 2) and the large area of the device.

In the Spray_HSV and Spray_SV films, which have similar nano-scale and meso-scale PCBM aggregation, the P3HT crystallization of the Spray_HSV film is much higher than that of the Spray_SV film (as evidenced by WAXS profiles). Thus, the hot solvent vapor annealing significantly improves PCE compared to RT solvent vapor annealing. From Table 2, for the device area of 1 × 0.3 cm^2^, the PCE of the device based on the Spray_HSV film is improved to 2.4%. Note that the main contribution is from the fill factor of 58.5%, which is higher than those of the devices based on the Spray_SV and Spray_T films (~43%). This result can be attributed to a significant increase in shunt resistance, R_sh_, (unit: Ωcm^2^)^12^. The increase in R_sh_ indicates that hot solvent vapor annealing reduces recombination and current leakage by (1) improving the interfacial contact and (2) forming a favorable transport path resulting from the spatial distribution of more P3HT crystallites, as shown in Fig. 2. The interpretation of GIWAXS and GISAXS analyses also supports this speculation. Moreover, when the device area increases to 1 × 1 cm^2^, the PCE of the device based on the Spray_HSV film is slightly reduced because of its relatively high J_SC_. This reduction may be attributed to the favorable mutual contact and spatial distributions of P3HT crystallites and PCBM clusters on the larger scale. The PCE of the device based on the Spray_SV + T film was improved to 2.2% by the PCBM aggregation and P3HT crystallization. The performance of this device is similar (high fill factor of 50.7% at the area of 1 × 0.3 cm^2^; high J_SC_ at the area of 1 × 1 cm^2^) to that of devices based on the Spray_SV film. The same explanation can be applied here.

The highest PCE (3.7%) is achieved for the device based on the Spray_HSV + T film. The increase in PCE (from 2.4% to 3.7%) produced by thermal annealing following hot solvent vapor annealing is mainly contributed by the P3HT crystallization. Although the redistribution and aggregation of PCBM clusters (governing the electron transport) is mainly tailored by the hot or RT solvent vapor annealing, the enhancement of polymer crystallization by further thermal annealing could be mutually coupled with the spatial distribution of the clusters to further improve the PCE. The polymer crystallization in this step optimizes the charge carrier transport and separation. The spin- and spray-coated PSCs have different BHJ structures (phase-separated P3HT and PCBM phases), although they are at the same thickness, leading to different performances. The discrepancy between the two BHJ structures is due to the existence of droplet boundaries (for the spray-coated film). The present study investigated spin- and spray-coated PSCs under their different optimum thicknesses. The variation in thickness for the spray-coated PSCs results in a change in performance. Our data show that increases in the durations of the post-treatments cannot significantly change the performance of the spray-coated PSCs (the performance corresponding to the spray-coated devices of different thickness and post-treatment parameter is in the Supplementary Information). The PL quenching for all films is consistent with the photovoltaics performance and the interpretation of charge transport network discussed in this present study. The effect of interfacial area cannot be closely correlated to the PL measurement (in the Supplementary Information).

## Discussion

The hierarchical BHJ structure, comprising (1) nano-scale edge-on and face-on P3HT crystallites with (100) lamellar structure, (2) nano-scale PCBM clusters and (3) the meso-scale structure of amorphous P3HT/PCBM domains, for the spray-processed active layers is revealed by GISAXS and GIWAXS characterization for the first time. The phase-separation mechanism tailored by thermal annealing, solvent vapor annealing and the combination of both is discussed here. These findings for the spray-coated films show substantial differences from those for the spin-coated blend films. The results of this structural characterization can provide insight into how the different types of processing can tailor different-scale structures. The correlation of the structural characterization with the performance of the OPV devices shows that networks of nanoscale PCBM clusters and P3HT crystallites play important roles when the OPV device area increases to 1 × 1 cm^2^ from 1 × 0.3 cm^2^. The formation and control of these networks, which are favorable for the transverse transport of electrons and holes, are closely related to the structure and aggregation distribution of droplet boundaries. Manipulating the droplet boundaries by hot solvent vapor and thermal annealing controls the effective re-distribution and growth (or aggregation) and 3D interpenetrating network of PCBM clusters and P3HT crystallites. The GISAXS/GIWAXS analysis also reveals the mutual contact or confinement between PCBM clusters and P3HT crystallites, serving the available role of charge carrier separation.

## Methods

### Preparation of blend films and devices

A total of 1 mg of P3HT and 1 mg of PCBM were dissolved in 1 ml of CB solvent. Six P3HT/PCBM blend films were prepared using the conventional spin-coating process. Then, five as-cast films were treated by (1) thermal annealing at 130 °C for 10 min, (2) solvent vapor annealing at room-temperature (RT) for 1 h, (3) hot solvent vapor annealing at 55 °C for 2 min, (4) RT solvent vapor plus thermal annealing (i.e., two steps combining (2) and then (1)) and (5) hot solvent vapor plus thermal annealing (i.e., combination of steps (3) and (1)). The treated films were called Spin_T, Spin_SV, Spin_HSV, Spin_SV + T and Spin_HSV + T, respectively. The as-cast spin-coated film was called Spin_C. These post-treatment procedures were described previously^12^. The thicknesses of the spin- and spray- coated blend films measured by the Alpha stepper were ~100 and ~250 nm, respectively. Six spray-coated films were treated with the same procedures as the spin-coated films; these are referred to as Spray_C, Spray_T, Spray_SV, Spray_HSV, Spray_SV + T and Spray_HSV + T. The corresponding OPV devices based on these sprayed films were fabricated with a structured ITO glass/PEDOT:PSS/BHJ active layer/Ca/Al. Voltage-photocurrent curves of these devices were measured under A.M. 1.5 illumination (100 mW/cm^2^) using a solar simulator (Abet technologies, Model #11000). All spray- and spin-coated samples for all tests were fabricated in the same bath. The device area is defined by the area of the Ca/Al electrode thermally deposited on the active layer. The prepared devices have areas of 1 × 0.3 and 1 × 1 cm^2^. Atomic force microscopy (AFM) was employed to study the BHJ morphology of the active layers. UV-visible absorption spectra were also measured for all films fabricated by the spraying process.

### Simultaneous GISAXS/GIWAXS experiment

Simultaneous GISAXS and GIWAXS measurement^16^,^17^,^19^ was performed at beam-line 23 A of the National Synchrotron Radiation Research Center, Taiwan, for all spin- and spray-coated films with the aforementioned post-treatments. The thin films for the GISAXS and GIWAXS experiments corresponding to the above processing conditions were deposited on silicon wafers (1 × 2 cm^2^) in the same batch. Our previous studies showed that the bulk morphologies of the active layers prepared on silicon and Si/PEDOT:PSS substrates and the corresponding GISAXS/GIWAXS intensities is almost the same (see the Supplementary Information of Ref. 16). The incident angle of the GISAXS/GIWAXS measurement to each thin film was precisely aligned to be 0.2 ± 0.002°, to penetrate the whole active layer^26^. The 2D GISAXS and GIWAXS patterns were simultaneously collected from two 2D detectors at different positions. The GISAXS profiles as a function of scattering vector *Q*_*x*_ were reduced from the 2D GISAXS patterns along the in-plane direction (parallel to the substrate or film surface; defined as the *X* direction). The out-of-plane GIWAXS profiles were reduced from the 2D GIWAXS patterns along the out-of-plane direction (perpendicular to the substrate and film surface; defined as the *Z* direction) and are expressed as a function of scattering vector *Q*_*z*_. The in-plane GIWAXS profiles as a function of *Q*_*x*_ were reduced along the in-plane direction of 2D GIWAXS pattern.

Essentially, the nano-scale P3HT crystallites in the film have two main orientations: (1) edge-on crystallites with a (100) lamellar layer parallel to the film surface; (2) face-on crystallites with a lamellar layer perpendicular to the film surface^13^,^29^. The orientation of face-on and edge-on P3HT crystallites has a slightly angular distribution (see the bright arc-like spots in the 2D GIWAXS pattern). There exist few P3HT crystallites with isotropic orientation, as shown in the isotropic ring in the 2D GIWAXS pattern. The position, width and area of (100) peak in the out-of-plane GIWAXS profiles reveal the spacing between (100) layers, the size of the crystalline domain and the relative crystallinity of edge-on P3HT crystallites, respectively. The in-plane WAXS profiles similarly reveal information regarding “face-on” P3HT crystals. The present work also integrated the peak intensity along the ring distribution to estimate the relative fraction in crystallinity of all oriented P3HT crystallites.

## Additional Information

**How to cite this article**: Huang, Y.-C. *et al.* Correlation between Hierarchical Structure and Processing Control of Large-area Spray-coated Polymer Solar Cell toward High Performance. *Sci. Rep.* **6**, 20062; doi: 10.1038/srep20062 (2016).

## Supplementary Material

Supplementary Information

## Figures and Tables

**Table 1:** Structural parameters determined by SAXS model fitting.

Treatment	*η*(%)	*R*(nm)	*p*	*A*	*ξ*(nm)
Spin_C	0.8	3.2	0.36	3.2 × 10^−5^	39.8
Spin_T	18.0	8.7	0.42	6.0 × 10^−5^	66.1
Spin_SV	9.5	6.3	0.45	10.4 × 10^−5^	41.9
Spin_HSV	9.7	6.6	0.34	6.0 × 10^−5^	36.6
Spin_SV + T	16.2	7.9	0.48	6.3 × 10^−5^	65.0
Spin_HSV + T	15.9	8.1	0.34	6.0 × 10^−5^	156.2
Spray_C	6.7	4.2	0.55	2.6 × 10^−5^	39.6
Spray_T	7.0	7.7	0.26	2.2 × 10^−5^	9.2
Spray_SV	12.5	8.7	0.42	9.0 × 10^−5^	35.6
Spray_HSV	12.8	7.6	0.51	9.0 × 10^−5^	33.7
Spray_SV + T	14.4	10.1	0.37	20.0 × 10^−5^	9.3
Spray_HSV + T	12.6	9.2	0.23	60.0 × 10^−5^	9.1

**Table 2:** Performance of the devices (device area = 1 × 0.3 cm^2^) based on the spray-coated films with various post-treatments.

Treatment	J_sc_ (mA/cm^2^)	V_oc_ (V)	FF (%)	η (%)	R_s_ (Ωcm^2^)	R_sh_ (Ωcm^2^)
C	4.515 ± 0.156 (4.66)	0.65 ± 0.002 (0.65)	44.85 ± 0.622 (45.4)	1.325 ± 0.083 (1.4)	16.45	415.47
T	6.446 ± 0.134 (6.59)	0.618 ± 0.002 (0.62)	42 ± 0.946 (43)	1.675 ± 0.083 (1.8)	13.39	311.70
SV	6.433 ± 0.077 (6.50)	0.61 ± 0.002 (0.61)	43.55 ± 0.269 (44)	1.7 ± 0 (1.7)	13.71	306.06
HSV	6.633 ± 0.125 6.567	0.625 ± 0.005 0.627	54.833 ± 4.299 58.5	2.267 ± 0.125 (2.4)	15.07	864.10
SV + T	7.329 ± 0.338 (6.87)	0.613 ± 0.007 (0.62)	46.167 ± 3.598 (50.7)	2.1 ± 0.082 (2.2)	15.41	1097.27
HSV + T	8.875 ± 0.078 (8.96)	0.635 ± 0.001 (0.64)	63.775 ± 1.008 (65.10)	3.6 ± 0.071 (3.73)	5.30	1035.76

These data averaged over 15 devices per processing condition. The highest performance was shown in bracket.

**Table 3:** Performance of the devices (device area = 1 × 1 cm^2^) based on the spray-coated films with various post-treatments.

Treatment	J_sc_ (mA/cm^2^)	V_oc_ (V)	FF (%)	η (%)
C	3.344 ± 0.221 (3.57)	0.609 ± 0.008 (0.62)	40.75 ± 0.25 (40.5)	0.835 ± 0.065 (0.9)
T	7.071 ± 0.024 (7.05)	0.598 ± 0.001 (0.60)	34.95 ± 0.25 (34.7)	1.5 ± 0 (1.5)
SV	5.274 ± 0.059 (5.33)	0.555 ± 0.007 (0.56)	30.85 ± 0.15 (30.7)	0.893 ± 0.007 (0.9)
HSV	7.791 ± 0.364 (7.43)	0.588 ± 0.01 (0.60)	48.35 ± 2.15 (50.5)	2.2 ± 0 (2.2)
SV + T	8.218 ± 0.102 (8.12)	0.587 ± 0.002 (0.59)	42.6 ± 0.8 (43.4)	2.05 ± 0.05 (2.1)
HSV + T	9.437 ± 0.475 (8.96)	0.6 ± 0 (0.6)	51.05 ± 4.75 (55.8)	2.9 ± 0.1 (3)

These data averaged over 15 devices per processing condition. The highest performance was shown in bracket.
