# Supplementary material for: Correlation between Hierarchical Structure and Processing Control of Large-area Spray-coated Polymer Solar Cells toward High Performance
Source: Sci Rep. 2016 Jan 28;6:20062. doi: 10.1038/srep20062 (PMC4730244; doi:10.1038/srep20062)
Supplement: Supplementary Information [file srep20062-s1.doc]

Supplementary Information

Correlation between Hierarchical Structure and Processing Control of Large-area Spray-coated Polymer Solar Cell toward High Performance

Yu-Ching Huang^1*^, Cheng-Si Tsao^1*^, Hou-Chin Cha^1^, Chih-Min Chuang^1^, Chun-Jen Su^2^, U-Ser Jeng^2^, Charn-Ying Chen^1^


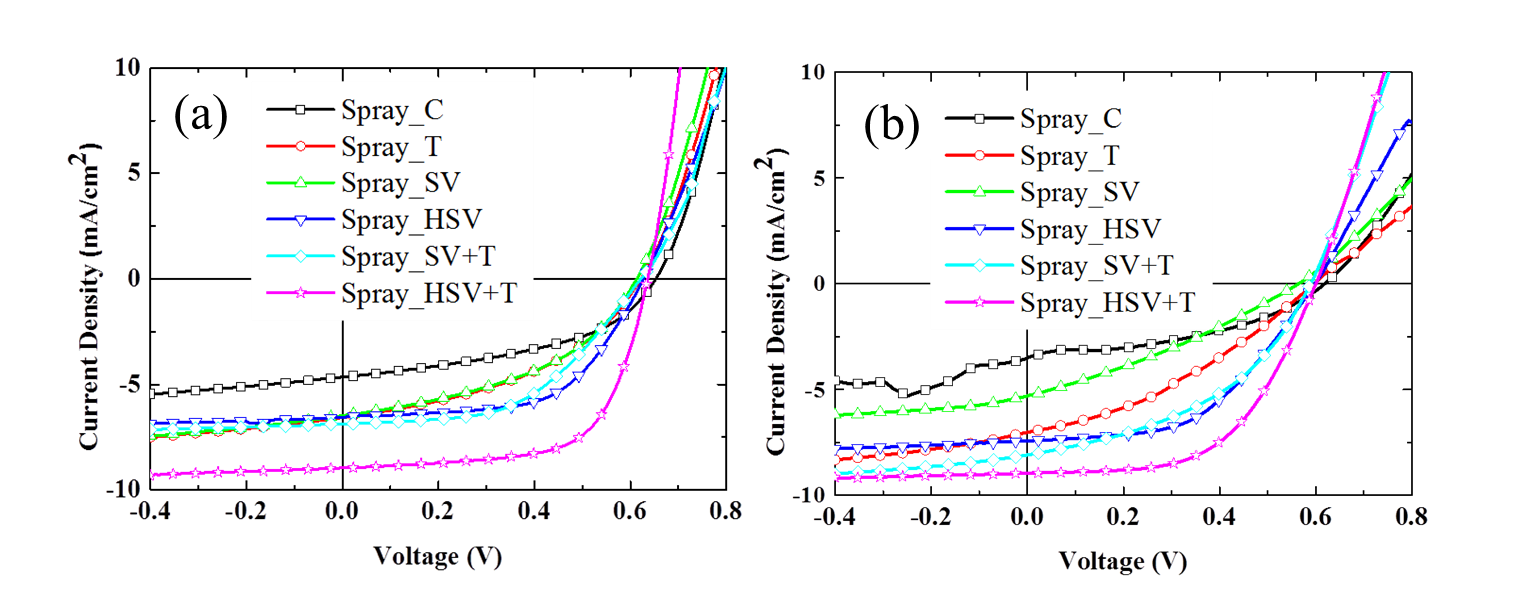


Figure S1. J-V curves of the devices area of (a) 1×0.3 cm^2^ and (b) 1×1 cm^2^ based on the spray-coated films with various post treatments.


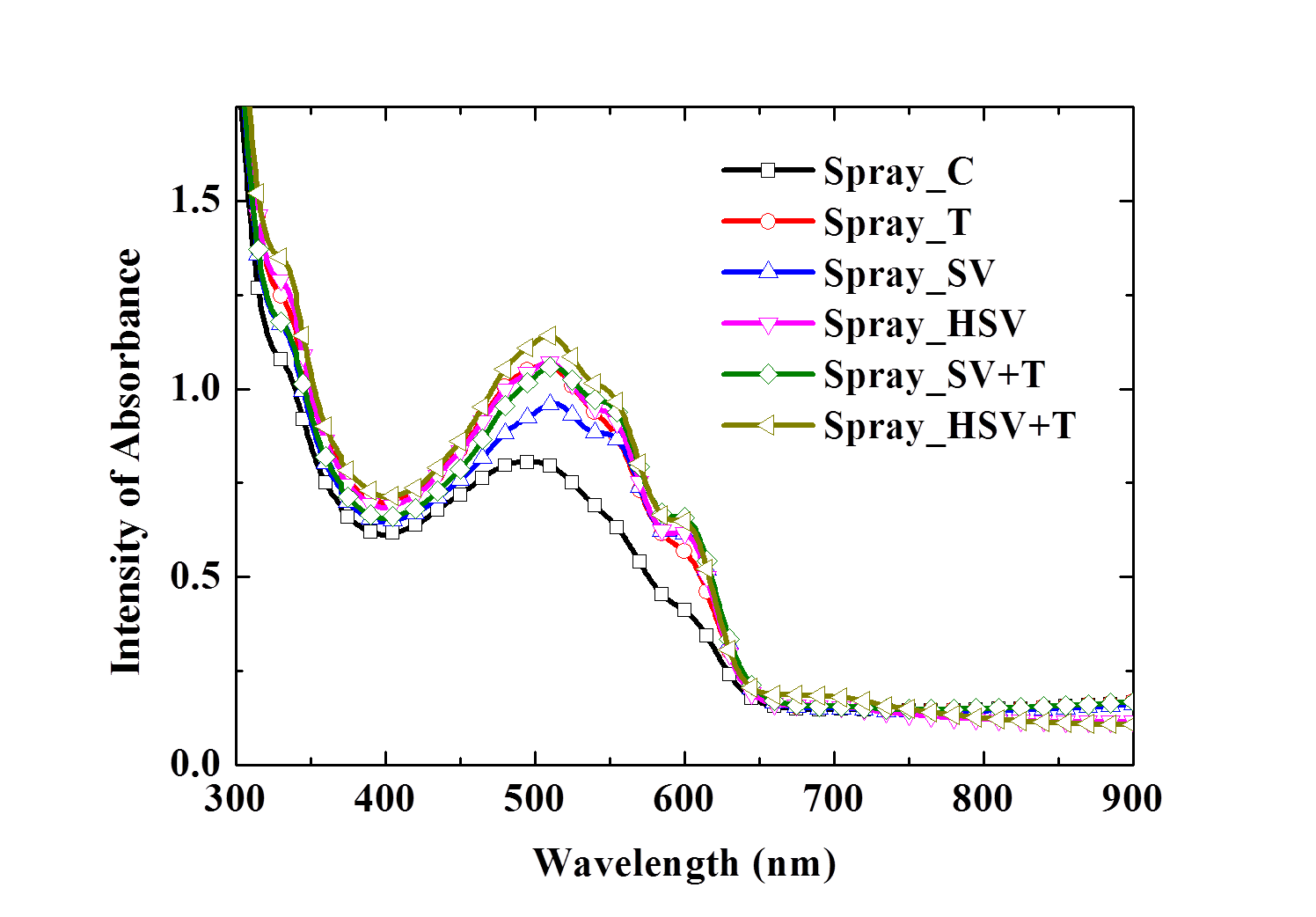


Figure S2. Absorption spectra of the spray-coated films with various post treatments.

Figure S3. PL characterization of the spray-coated films with different treatments.

Table S1 Performance of the devices (device area = 1×0.3 cm^2^) based on the spin-coated films with various post-treatments. These data averaged over 15 devices per processing condition. The highest performance was shown in bracket.

| Treatment | J_sc_ (mA/cm^2^) | V_oc_ (V) | FF (%) | η (%) |
| --- | --- | --- | --- | --- |
| C | 8.517±0.367  (8.45) | 0.502±0.009  (0.51) | 42.167±1.658  (44.5) | 1.8±0.082  (1.9) |
| T | 10.663±0.081  (10.64) | 0.595±0.002  (0.59) | 59.75±2.339  (62.7) | 3.8±0.158  (4) |
| SV | 8.005±0.187  (7.99) | 0.561±0.007  (0.55) | 61.033±1.893  (63.7) | 2.733±0.094  (2.8) |
| HSV | 6.375±0.033  (6.40) | 0.565±0.009  (0.56) | 60.467±1.382  (61.9) | 2.167±0.047  (2.2) |
| SV+T | 9.648±0.306  (9.48) | 0.573±0.007  (0.56) | 60.967±2.136  (63.4) | 3.367±0.047  (3.4) |
| HSV+T | 9.165±0.639  (9.47) | 0.577±0.012  (0.59) | 64.133±1.94  (65.3) | 3.4±0.216  (3.6) |

Table S2 Performance of the spray-coated devices with two thicknesses of 100 and 250 nm, respectively, processed with different post-treatments.These data averaged over 5 devices per processing condition. The highest performance was shown in bracket.

| Treatment | J_sc_ (mA/cm^2^) | V_oc_ (V) | FF (%) | η (%) |
| --- | --- | --- | --- | --- |
| C  100 nm | 2.293±0.131  (2.29) | 0.572±0.012  (0.58) | 33.6±0.731  (34.3) | 0.45±0.05  (0.5) |
| SV  100 nm | 4.92±0.036  (4.96) | 0.49±0.016  (0.48) | 30.375±1.295  (32.6) | 0.75±0.05  (0.8) |
| SV  250 nm | 6.433±0.077  (6.50) | 0.61±0.002  (0.61) | 43.55±0.269  (44) | 1.7±0  (1.7) |
| HSV  100 nm | 6.566±0.013  (6.57) | 0.562±0.007  (0.57) | 38.3±0.851  (39.5) | 1.425±0.083  (1.5) |
| HSV  250 nm | 6.633±0.125  (6.57) | 0.625±0.005  (0.63) | 54.833±4.299  (58.5) | 2.267±0.125  (2.4) |

Table S3 Performances of the spray-coated devices with thermal and solvent vapor treatment for the different annealing times. These data averaged over 5 devices per processing condition. The highest performance was shown in bracket.

| Treatment | J_sc_ (mA/cm^2^) | V_oc_ (V) | FF (%) | η (%) |
| --- | --- | --- | --- | --- |
| T-10 min | 6.446±0.134  (6.59) | 0.618±0.002  (0.62) | 42±0.946  (43) | 1.675±0.083  (1.8) |
| T-30 min | 5.755±0.328  (5.59) | 0.613±0.01  (0.62) | 49.275±3.556  (53.6) | 1.75±0.112  (1.9) |
| SV-1 h | 6.433±0.077  (6.50) | 0.61±0.002  (0.61) | 43.55±0.269  (44) | 1.7±0  (1.7) |
| SV-2 h | 5.895±0.928  (5.44) | 0.59±0.05  (0.62) | 47.775±3.043  (51.6) | 1.625±0.083  (1.7) |
